# Supplementary material for: Development and Validation of an Environmental Health Literacy Assessment Screening Tool for Domestic Well Owners: The Water Environmental Literacy Level Scale (WELLS)
Source: Int J Environ Res Public Health. 2019 Mar 11;16(5):881. doi: 10.3390/ijerph16050881 (PMC6427415; doi:10.3390/ijerph16050881)
Supplement: Supplementary file 1 [file ijerph-16-00881-s001.pdf]

Supplementary Information

Development and Validation of an Environmental Health Literacy Assessment Screening Tool for Domestic Well Owners: The Water Environmental Literacy Level Scale (WELLS)

Veronica L. Irvin *, Diana Rohlman, Amelia Vaughan, Rebecca Amantia, Claire Berlin and Molly L. Kile

College of Public Health and Human Sciences, Oregon State University, Corvallis, OR 97330, USA; Diana.Rohlman@oregonstate.edu (D.R.); Amelia.Vaughan@oregonstate.edu (A.V.); Rebecca.Amantia@oregonstate.edu (R.A.); Berlinc@oregonstate.edu (C.B.); Molly.Kile@oregonstate.edu (M.L.K.)

* Correspondence: Veronica.Irvin@oregonstate.edu

Table S1. Specific wording and distribution for question and response option for the items in the Water Environmental Literacy Level scale as measured with an online adult sample, 2016

Below please see the table describing how to interpret results from testing household water for arsenic, a heavy metal. Please answer the following questions based on information available in this table.		N (%)
How many ppb of arsenic is safe for cooking?		
a.	8	844 (97.1%)
b.	15	5 (0.6%)
c.	50	10 (1.2%)
d.	100	10 (1.2%)
How many ppb of arsenic in water is safe for domestic use?		
a.	450	43 (5.0%)
b.	99	278 (32.0%)
c.	Both a & b	354 (40.7%)
d.	Neither a nor b	194 (22.3%)
Your well water test reports that your well water is not safe for drinking. What can you do?		
a.	Boil water	35 (4.0%)
b.	Drink bottled water	818 (94.4%)
c.	Ignore it	1 (0.1%)
d.	Test well water every 5 years	13 (1.5%)
Your water testing result shows arsenic at 50 ppb. How many millligrams (mg) is in one liter of your water?		
a.	.05	668 (77.0%)
b.	.5	138 (15.9%)
c.	5	31 (3.6%)
d.	50	15 (3.6%)
Pretend that your household water contains 15ppb of arsenic. Is it safe for you and your pets to drink?		
a.	No	832 (95.7%)
a.	Not enough information to answer this question	12 (1.4%)

b. Not for me, but it is safe for my pet	6 (0.7%)
c. Yes, both my pet and me	19 (2.2%)
If no, why not? Open-ended responses must have discussed some variant of contaminant level in table was linked to not being safe for drinking or by pets.	
a. Incorrect response	44 (5%)
b. Correct response	825 (95%)

Table S2. Specific wording and distribution for question and response option for the items in the Newest Vital Sign scale as measured with an online adult sample, 2016.

**Please see the sample ice cream nutritional label and answer the following questions based on information only available in this figure.**	**N (%)**
If you eat the entire container, how many calories will you eat?	
a. 250	19 (2.2%)
b. 500	12 (1.4%)
c. 750	1 (0.1%)
d. 1000	836 (96.3%)
If you are allowed to eat 60 grams of carbohydrates as a snack, how much ice cream could you have?	
e. 1 cup	296 (34.1)
f. Half the container	115 (13.2%)
g. Both a & b	440 (50.6%)
h. Neither a nor b	18 (2.1%)
Your doctor asks you to reduce the amount of saturated fat in your diet. You usually have 42 g of saturated fat each day, which includes 1 serving of ice cream. If you stop eating ice cream, how many grams of saturated fat would you be consuming each day?	
e. 9	56 (6.5%)
f. 24	26 (3.0%)
g. 33	753 (86.9%)
h. 42	32 (3.7%)
If you usually eat 2,500 calories in a day, what percentage of your daily value of calories will you be eating if you eat one serving?	
e. 5%	36 (4.1%)
f. 10%	773 (89.0%)
g. 20%	47 (5.4%)
h. 50%	13 (1.5%)
For the next two questions, pretend that you are allergic to the following substances: penicillin, peanuts, latex gloves, and bee stings. Is it safe for you to eat this ice cream?	
d. Yes	95 (10.9%)
e. No	774 (89.1%)
If no, why not? Open-ended responses must have discussed peanut allergy in some context to be scored correct.	
c. Incorrect response	102 (12%)
d. Correct response	764 (88%)
